# Challenging the myth: comparing early complications of native and periprosthetic distal femur fractures. The role of implant stability

**DOI:** 10.1007/s00402-025-06173-4

**Published:** 2026-01-07

**Authors:** Christopher Lampert, Leon Faust, Gautier Beckers, Adrian Cavalcanti Kußmaul, Boris Michael Holzapfel, Wolfgang Böcker, Carl Neuerburg, Florian Pachmann

**Affiliations:** Department of Orthopaedics and Trauma Surgery, Musculoskeletal University Center Munich (MUM), LMU Klinikum, Munich, Germany

**Keywords:** Periprosthetic fractures, Arthroplasty, Internal fixation, Reoperation, Femoral fractures, Postoperative complications

## Abstract

**Introduction:**

Periprosthetic distal femur fractures are often considered more complex and morbid than native distal femur fractures, yet few studies directly compare them. This study aimed to compare patient characteristics, treatment strategies, and early complications between native and periprosthetic distal femur fractures using the Lewis–Rorabeck classification.

**Methods:**

We retrospectively analyzed 152 patients treated surgically for distal femur fractures at a level I trauma center. Cases included native fractures (*n* = 90) and periprosthetic distal femur fractures (*n* = 62), further divided into Lewis–Rorabeck Type I/II (*n* = 49) and Type III (*n* = 13). Demographics, comorbidities, surgical details, and in-hospital complications were assessed. We conducted a multivariate analysis comparing native fractures with Lewis–Rorabeck Type I/II fractures, as well as comparing Type I/II with Type III fractures.

**Results:**

Patients with periprosthetic fractures were older and had higher BMI than those with native fractures (*p* < 0.001). Multivariate analysis showed no significant differences in surgery duration, mortality, mobility at discharge, transfusion needs, or revision rates between native distal femur fractures (AO/OTA Type A) and Lewis–Rorabeck Type I/II. The complication rate was significantly lower in the periprosthetic group (*p* = 0.029). Lewis–Rorabeck Type III fractures showed significantly longer time to surgery (*p* = 0.015) and revision surgery was performed more frequently compared to Lewis–Rorabeck Type I/II fractures. However, no differences were observed in early postoperative complications, mobility at discharge, or length of hospital stay.

**Conclusion:**

This study directly compares periprosthetic distal femur fractures stratified by implant stability to native distal femur fractures. The findings challenge the perception that periprosthetic fractures are universally more difficult to treat. Periprosthetic fractures achieve results comparable to native fractures. The increased complexity of Lewis–Rorabeck Type III fractures is reflected in prolonged time to surgery and an increased need for revision surgery.

**Supplementary Information:**

The online version contains supplementary material available at 10.1007/s00402-025-06173-4.

## Introduction

The increasing number of total joint arthroplasties performed annually, driven by an aging population and rising demand for improved quality of life post-arthroplasty, have contributed to an elevated risk of periprosthetic fractures (PPFs) [1]. Despite their rarity, PPFs are a serious complication and have been reported as the third leading cause for revision surgery, after aseptic loosening and recurrent dislocation [2, 3].

Distal femur fractures can occur either in the surrounding of an endoprosthesis or affect the native distal femur. PPFs of the distal femur following total knee arthroplasty (TKA) occur in approximately 2.5% of TKA cases [4]. While its native counterpart also account only for a relatively small percentage of all femoral fractures, both fracture types are commonly associated with substantial morbidity, elevated mortality rates, and poor surgical outcomes including limited mobility, particularly in elderly patients with multiple comorbidities [5–8]. The management of native distal femur fractures is well-established, with a variety of osteosynthesis techniques guided by the Arbeitsgemeinschaft für Osteosynthesefragen/Orthopaedic Trauma Association (AO/OTA) classification system. However, these techniques are only partially applicable to periprosthetic fractures, leading to the development of the Lewis-Rorabeck classification [9]. This classification considers both the displacement of the fracture and the stability of the implant, which guide the surgical approach. Type I describes a nondisplaced fracture with a stable femoral component, Type II a displaced fracture with a stable component, and Type III a fracture associated with a loose femoral component. Open reduction and internal fixation (ORIF) is typically recommended for Lewis–Rorabeck Type I–II fractures with a stable component, whereas Type III fractures generally require revision arthroplasty due to component loosening [10, 11].

Despite the initial similarity between both native distal femur fractures and PPF, there is limited literature directly comparing these fracture types. Understanding the differences in patient demographics, fracture patterns, and early complications between these groups is crucial for optimizing treatment protocols. Since PPFs frequently occur in older and more vulnerable patients, it is essential to develop a nuanced approach and a comprehensive understanding of the different treatment strategies.

This retrospective study aims to compare native and periprosthetic distal femur fractures with respect to treatment approaches, perioperative complications, short-term functional outcomes, and early mortality. Furthermore, special consideration was given to the fracture configuration of native fractures and the stability of the prosthetic components.

## Materials and methods

This retrospective study was conducted at a level I trauma center in Germany. Patients aged 18 years and older who sustained either a distal femur fracture or a periprosthetic distal femur fracture post-TKA and received definitive surgical treatment between January 2017 and August 2023 were included in this study. In patients with bilateral distal femur fractures, each fracture was recorded as a separate case, while patient-related variables such as age, sex, and comorbidities were counted once per patient. Patients who received temporary treatment, such as external fixation or spacer implantation, and those with pathological fractures were excluded. Distal femur fractures were included regardless of AO/OTA classification to ensure an adequate sample size. Periprosthetic fractures following TKA were classified according to the Lewis-Rorabeck classification [9]. Lewis–Rorabeck Types I and II were grouped because both involve a stable component and are usually treated with ORIF. The study was conducted in accordance with the Declaration of Helsinki. Ethical approval was given by the local ethics committee (Ref.-No. 25–0704) and the study was registered in the German Clinical Trials Registry (DRKS ID: DRKS00038720).

Clinical data were extracted from the institutional electronic health record system, Meona (Meona GmbH, Freiburg, Germany). The extracted data were anonymized and entered into a Microsoft Excel spreadsheet (Microsoft Corporation, Redmond, WA, USA). Demographic variables included age, sex, and body mass index (BMI). Clinical variables comprised American Society of Anesthesiologists (ASA) classification and comorbidities. Data on fracture characteristics, mechanisms of injury, and type of surgical procedure were collected. Primary outcomes were inpatient mortality, early postoperative mobility (defined as the ability to mobilize out of bed and walk with or without a walking aid), and length of hospital stay. Secondary outcomes included time from admission to surgery, operative duration, blood product volume transfused, postoperative complications, and the need for revision surgery. Non-surgical complications comprised sepsis, pneumonia, urinary tract infections, pleural effusion, acute kidney injury, and pulmonary embolism. Surgical complications and revision procedures were assessed both during the initial hospitalisation and during the available follow-up period after discharge. The mean follow-up duration was 7.9 months (SD 12.9; range 1–72 months). A total of 72 patients (47.4%) were lost to follow-up. Revision surgeries occurred on average 4.1 months (SD 4.0) after the index procedure. To ensure a certain standard of quality, patients were treated by a board-certified orthopedic surgeon on the basis of standard operating procedures (SOPs). In native distal femur fractures, lateral fixation was performed using LCP Distal Femur Plates (DePuy Synthes), NCB Periprosthetic Femur Plates (Zimmer), Biphasic Plates Distal Femur (AO Research Institute), TOMOFIX Plates (DePuy Synthes), or VA-LCP Condylar Plates (DePuy Synthes). Medial fixation was achieved with LCP 3.5 mm (DePuy Synthes) or Pelvic Reconstruction Plates (DePuy Synthes), or TOMOFIX Plates (DePuy Synthes). In Lewis–Rorabeck Type I/II fractures, the same implant systems applied for lateral fixation in native fractures were used, namely the LCP Distal Femur Plates (DePuy Synthes), NCB Periprosthetic Femur Plates (Zimmer), Biphasic Plates Distal Femur (AO Research Institute), TOMOFIX Plates (DePuy Synthes), or VA-LCP Condylar Plates (DePuy Synthes). In Lewis–Rorabeck Type III fractures, fixation was performed using the VA-LCP Condylar Plates (DePuy Synthes).

Descriptive statistics were reported as frequencies, means, standard deviations (SD), and sample sizes (n). Normality of data distribution was assessed using the Kolmogorov-Smirnov and Shapiro-Wilk tests. For non-normally distributed and ordinal data, the Mann-Whitney U test was applied. Categorical variables were analyzed using either the Chi-square test or Fisher’s exact test, as appropriate based on sample size and expected cell counts. Parametric data were compared using the independent samples t-test. For comparisons involving more than two groups, the Kruskal-Wallis test was used for non-normally distributed data, while one-way ANOVA was employed for normally distributed data. All statistical tests were two-sided. To assess differences in early complications between the different fracture types linear and logistic regression models were employed. To control for potential confounders all multivariate analyses were adjusted for age, sex, BMI and ASA score. Results are presented as regression coefficients (β) for linear regression and odds ratios (OR) for logistic regression, with corresponding 95% confidence intervals (CI). P-value < 0.05 was considered statistically significant. All analyses were performed using IBM Statistical Package for the Social Sciences (SPSS) (IBM Corporation, Armonk, NY, USA).

## Results

A total of 148 patients with 152 fractures were included in the study, consisting of 90 native distal femur fractures and 62 periprosthetic distal femur fractures. Among the periprosthetic fractures, 49 fractures were classified as Lewis–Rorabeck Type I or II fractures, and 13 as Type III fractures.

Demographic analysis revealed statistically significant differences in age and BMI between the groups (*p* = 0.002 and *p* < 0.001). The mean age was lowest in patients with native distal femur fractures (71.57 ± 19.69 years) and highest in those with Lewis–Rorabeck Type I and II fractures (LR 1/2) (82.60 ± 10.15 years). Similarly, BMI was lowest in patients with native distal femur fractures (23.31 ± 4.97 kg/m²) and highest in patients with Lewis–Rorabeck Type III (LR 3) fractures (30.0 ± 11.63 kg/m²) (Table 1).

The analysis of preoperative health status revealed no significant differences in ASA classification. Patients with PPF tended to exhibit higher ASA scores compared to those with native distal femur fractures. Specifically, ASA Class III or higher was most frequently observed in patients with Lewis–Rorabeck Type I/II fractures (LR 1/2: 70.8% vs. LR3 61.5% vs. Native 57.5%). However, this difference did not reach statistical significance (*p* = 0.331). The comorbidity burden was high; hypertension (*p* = 0.001), atrial fibrillation (*p* = 0.038) and chronic heart failure (*p* = 0.020) were significantly more prevalent among patients with periprosthetic fractures.

**Table 1:** Baseline patient characteristics

	Total *n* = 148	Native distal femur fracture *n* = 87	Lewis-Rorabeck Type I/II *n* = 48	Lewis-Rorabeck Type III *n* = 13	*p*-value
Mean ± SD					
Age (years)	76.47 ± 16.75	71,57 ± 19.69	82.60 ± 10.15	80.69 ± 8.22	**0.002**
BMI (kg/m²)	24.95 ± 6.54	23.31 ± 4.97	26.54 ± 6.27	30.00 ± 11.63	**< 0.001**
Percent of patients (n)					
Sex (female)	75.0% (111)	69.0% (60)	85.4% (41)	76.9% (10)	0.106
Bilateral fracture	2.7% (4)	3.4% (3)	2.1% (1)		
ASA					0.331
I	2.7% (4)	4.6% (4)			
II	25.7% (38)	27.6% (24)	18.8% (9)	38.5% (5)	
III	62.2% (92)	57.5% (50)	70.8% (34)	61.5% (8)	
IV	9.5% (14)	10.3% (9)	10.4% (5)		
Low-energy trauma	71.6% (106)	74.7% (65)	66.7% (32)	69 − 2% (9)	0.599
Comorbidities					
Hypertension	50.7% (75)	33.3% (29)	75.0% (36)	76.9% (10)	**0.001**
Osteoporosis	29.1% (43)	29.9% (26)	31.3% (15)	15.4% (2)	0.556
AF	22.3% (33)	14.9% (13)	33.3% (16)	30.8% (4)	**0.038**
Dementia	16.9% (25)	16.1% (14)	20.8% (10)	7.7% (1)	0.531
T2DM	12.8% (19)	9.2% (8)	18.8% (9)	15.4% (2)	0.264
CKD	9.5% (14)	6.9% (6)	12.5% (6)	15.4% (2)	0.479
CHD	8.1% (12)	8.0% (7)	10.4% (5)		0.475
CHF	7.4% (11)	2.3% (2)	14.6% (6)	15.4% (2)	**0.020**
COPD	4.7% (7)	5.7% (5)	4.2% (2)		0.789

Values are presented as mean ± standard deviation or percentage unless otherwise indicated

*BMI* body mass index, *ASA* american society of anesthesiologists, *AF* atrial fibrillation, *T2DM* type-2 diabetes mellitus, *CKD* chronic kidney disease, *CHD* coronary heart disease, *CHF* chronic heart failure, *COPD* chronic obstructive pulmonary disease, *SD* standard deviation

Significant p values are in bold

**Fig. 1:**
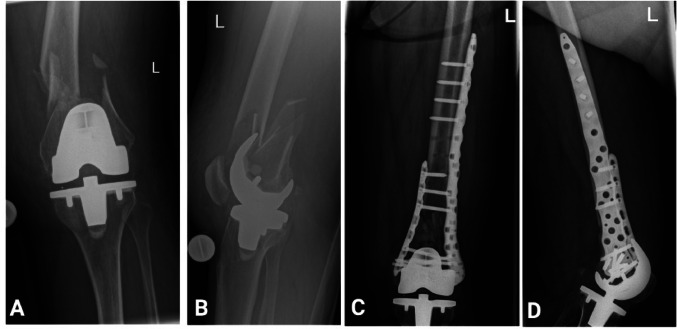
Case example of a Lewis–Rorabeck Type II periprosthetic distal femur fracture. Pre- and postoperative radiographs of a 72-year-old female patient with a Lewis–Rorabeck type II periprosthetic distal femur fracture sustained after a low-energy fall while gardening. **A** Preoperative anteroposterior and B lateral radiographs of the knee demonstrating the fracture. **C** Postoperative anteroposterior and **D** lateral radiographs following fixation with medial and lateral double plating, performed on the day of hospital admission

The majority of the patients with native distal femur fractures were treated with ORIF (92.2%, *n* = 83). Of these, 82.7% (*n* = 67) underwent single lateral plating, while 6.2% (*n* = 5) received dual plating with lateral and medial plates. ORIF was also the most common type of surgery for Lewis-Rorabeck Type I/II (95.9%, *n* = 47). Most of them also received single plating on the lateral side (97.6%, *n* = 40), with most patients receiving single lateral plating (97.6%, *n* = 40) and only one patient was treated with dual plating (Fig. 1).

**Fig. 2:**
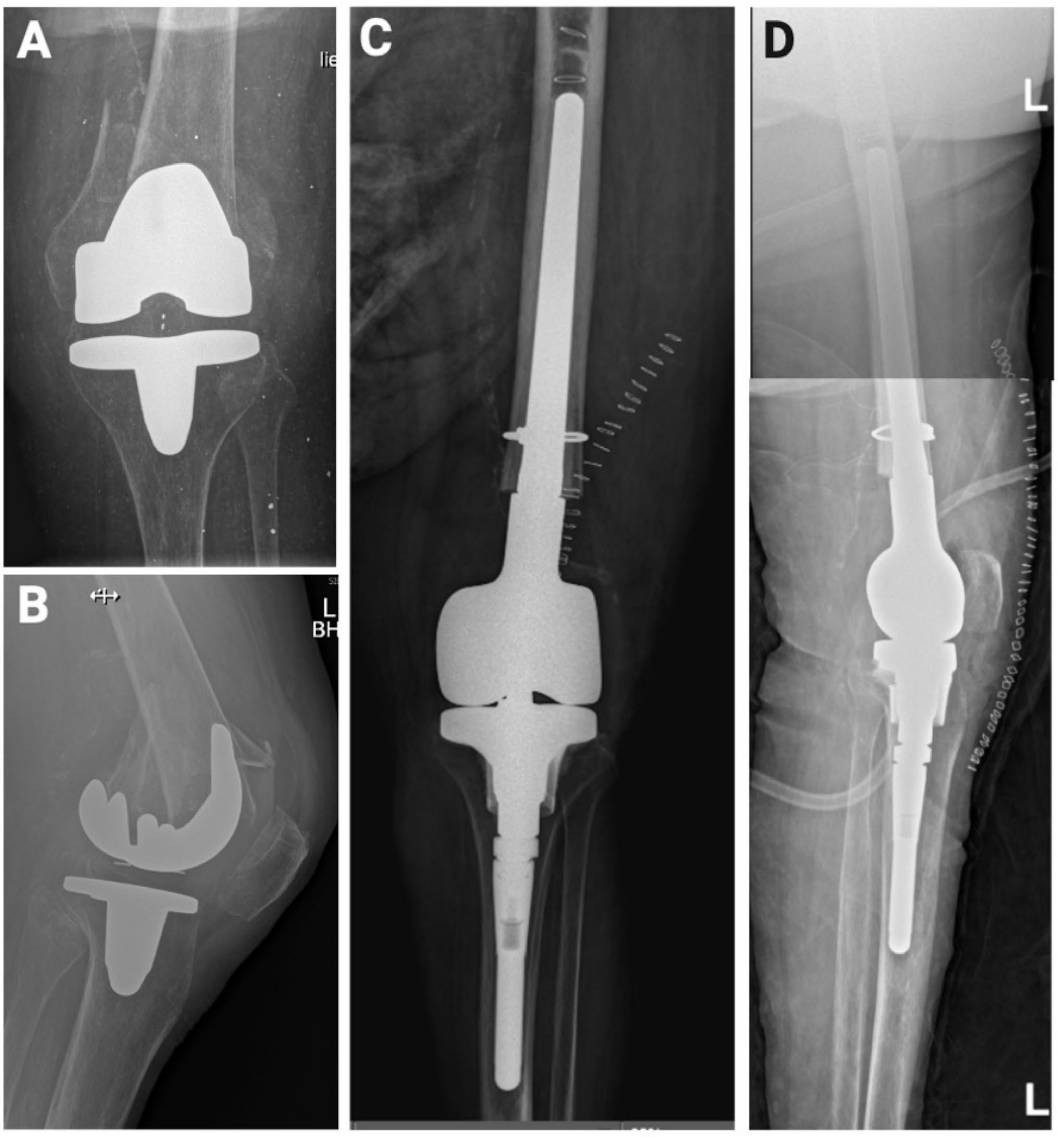
Case example of a Lewis–Rorabeck Type III periprosthetic distal femur fracture. Pre- and postoperative radiographs of an 84-year-old female patient with a Lewis–Rorabeck Type III periprosthetic distal femur fracture sustained after a fall from standing height. **A** Preoperative anteroposterior and **B** lateral radiographs of the knee demonstrating the fracture. **C** Postoperative anteroposterior and **D** lateral radiographs following distal femoral replacement, performed the day after hospital admission

In contrast, revision arthroplasty was the main procedure in Lewis–Rorabeck Type III fractures (92.3%, *n* = 12) (Fig. 2).

Among the Type III cases, 10 underwent distal femoral replacement (DFR) and 2 received revision-type implants. Postoperative weight-bearing protocols differed by fracture type, with most Type III patients allowed weight bearing as tolerated (84.6%), whereas partial weight-bearing was more common in Type I/II and native distal femur fractures (51.0% and 60.0%, respectively) (Table 2).

**Table 2:** Surgical procedure and postoperative mobilization protocols according to fracture type

Procedure		Native distal femur fracture	Lewis-Rorabeck Type I/II	Lewis-Rorabeck Type III	
Percent of cases (n)					
ORIF	Total	90.0% (81)	83.6% (41)	7.7% (1)	
	Screw fixation only	11.1% (9)	–	–	
	Single plating (lateral)	82.7% (67)	97.6% (40)	100% (1)	
	Double plating (medial + lateral)	6.2% (5)	2.4% (1)	–	
Intramedullary nailing		5.6% (5)	–	–	
Arthroplasty/Revision arthroplasty		4.4% (4)	16.4% (8)	92.3% (12)	
Postoperative weight bearing regime	WBAT	40.0% (36)	49.0% (24)	84.6% (11)	
	PWB	60.0% (54)	51.0% (25)	15.4% (2)	

Values are presented as percentage and number of cases

*ORIF* Open reduction internal fixation, *WBAT* weight bearing as tolerated, *PWB* partial weight bearing

Table 3 summarizes all surgical and non-surgical complications following definitive treatment. Urinary tract infection was the most frequent non-surgical complication, followed by pneumonia and pleural effusion. Surgical complications varied by treatment strategy, with periprosthetic infections being more common after treatment of Lewis–Rorabeck Type III fractures. Wound healing disorders dominated in native distal femur fractures. Non unions were only observed in Lewis-Rorabeck Type I/II Fractures.

**Table 3:** Overview of surgical and non-surgical complications after definitive surgery

		Native distal femur fracture	Lewis-Rorabeck Type I/II	Lewis-Rorabeck Type III	*p*-value
		*n* = 90	*n* = 49	*n* = 13	
Percent of cases (n)					
Non-surgical complications	Urinary tract infection	11.11% (10)	8.16% (4)	7.69% (1)	0.825
	Pneumonia	6.67% (6)	2.04% (1)	–	0.328
	Pleural effusion	4.44% (4)	4.08% (2)	–	0.743
	Acute kidney injury	2.22% (2)	2.04% (1)	15.38% (2)	**0.038**
	Lung artery embolism	3.33% (3)	–	–	0.348
	Sepsis	1.11% (1)	–	–	0.707
Surgical complications	Periimplant/periprosthetic infection	1.11% (1)	2.04% (1)	7.69% (1)	0.280
	Wound healing disorder	3.33% (3)	–	–	0.348
	Non-union	–	4.08% (2)	–	0.119
	Secondary dislocation	1.11% (1)	–	–	0.707
	Implant loosening	–	–	15.38% (2)	**–**
	Revision Surgery performed	6.67% (6)	6.12% (3)	23.08% (3)	0.104

All surgical and non-surgical complications are reported in this table. Individual patients may have experienced more than one complication, and each complication was recorded separately.

When comparing early complications between patients with distal femur fractures (AO/OTA Type A) and those with Lewis–Rorabeck Type I/II periprosthetic fractures, the analysis revealed a significantly lower complication rate in the periprosthetic fracture group compared to the distal femur fracture group (14.29% vs. 30.61%, *p* = 0.029). No statistically significant differences were observed in operative duration (125.35 ± 49.18 min vs. 148.02 ± 72.72 min, *p* = 0.310) or time to surgery (27.97 ± 33.70 h vs. 30.74 ± 29.98 h, *p* = 0.421) between the two groups. While patients with Lewis–Rorabeck Type I/II fractures had a shorter mean length of hospital stay and walking ability at discharge appeared slightly better in the periprosthetic group compared to those with native distal femur fractures (17.82 ± 13.56 days vs. 13.29 ± 5.58 days), this difference did not reach statistical significance (*p* = 0.252. No significant differences were observed between the groups in terms of mortality, the need for blood transfusion, the rate of revision surgery, or walking ability at discharge (Table 4). When comparing all distal femur fractures (AO/OTA Types A–C) with Lewis–Rorabeck Type I/II periprosthetic fractures, no significant differences were found for any of these parameters (Supp. 1).

**Table 4:** Analysis of Lewis-Rorabeck type I/II and native distal femur fractures type A on perioperative parameters and early complications

	Native distal femur fracture Type A (*n* = 49)	Lewis-Rorabeck Type I/II (*n* = 49)	β/OR	*p*-value
Mean ± SD				
Duration of surgery (minutes)	125.35 ± 49.18	148.02 ± 72.72	– 14.46	0.310
Time to surgery (hours)	27.97 ± 33.70	30.74 ± 29.98	– 5.98	0.421
Hospital stay (days)	17.82 ± 13.56	13.29 ± 5.58	2.68	0.252
Percent of cases (n)				
Patients with ≥ 1 non-surgical complication	30.61% (15)	14.29% (7)	3.77	**0.029**
Inpatient mortality	6.12% (3)	4.08% (2)	11.73	0.109
Patients receiving blood transfusion	20.41% (10)	28.57% (14)	0.80	0.703
Revision surgery performed	10.20% (5)	6.12% (3)	1.40	0.705
Walking ability at discharge	51.02% (25)	61.22% (30)	2.32	0.950

Values are presented as mean ± standard deviation or percentage unless otherwise indicated

*n* numbers of observation, *β* Regression Coefficient, *OR* Odds Ratio, *SD* standard deviation, All models were adjusted for sex, age, BMI and ASA Score

Significant p values are in bold

A comparison between Lewis–Rorabeck Type I/II and Type III fractures revealed significant differences in time to primary surgery (30.74 ± 29.98 h vs. 70.23 ± 92.43 h, *p* = 0.015). However, this did not result in any difference in the length of hospital stay (13.29 ± 5.58 days vs. 15.54 ± 3.53 days, *p* = 0.196). Revision surgeries occurred more frequently in Lewis–Rorabeck Type III fractures, although this difference did not reach statistical significance (6.12% vs. 23.08%, *p* = 0.081). Operative duration, in-hospital mortality, transfusion requirements, revision surgery, and walking ability were comparable between the two groups (Table 5).

**Table 5:** Comparison between Lewis-Roraback I/II and Lewis-Rorabeck III fractures on perioperative parameters and early complications

		Lewis-Rorabeck Type I/II (*n* = 49)	Lewis-Rorabeck Type III (*n* = 13)	β/OR	*p*-value
Mean ± SD					
Duration of surgery (minutes)		148.02 ± 72.72	134.85 ± 33.49	9.19	0.676
Time to surgery (hours)		30.74 ± 29.98	70.23 ± 92.43	– 40.94	**0.015**
Hospital stay (days)		13.29 ± 5.58	15.54 ± 3.53	– 2.15	0.196
Percent of cases (n)					
Patients with ≥ 1 non-surgical complication		14.29% (7)	15.38% (2)	0.440	0.413
Inpatient mortality		4.08% (2)	7.69% (1)	0.213	0.315
Patients receiving blood transfusion		28.57% (14)	38.46% (5)	0.335	0.159
Revision surgery performed		6.12% (3)	23.08% (3)	0.124	0.081
Walking ability at discharge		61.22% (30)	69.23 (9)	0.895	0.883

Values are presented as mean ± standard deviation or percentage unless otherwise indicated

*n* numbers of observation, *β* regression coefficient, *OR* odds ratio, *SD* standard deviation, All models were adjusted for sex, age, BMI and ASA Score

Significant p values are in bold

## Discussion

A key finding of this analysis was that periprosthetic distal femur fractures with a stable implant (Lewis-Rorabeck Type I/II) had a similar early clinical course to native distal femur fractures in terms of inhospital mortality, the need for revision surgery, length of hospital stay, and mobility at discharge. Interestingly, the postoperative complication rate was lower in the periprosthetic group.

Although several clinical series have examined outcomes following periprosthetic distal femur fractures, only a limited number of studies have directly compared these injuries with native distal femur fractures. Notably, none of the previous studies have distinguished between different types of periprosthetic fractures or accounted for the specific surgical procedures used (e.g., ORIF vs. revision arthroplasty) when comparing them with their native counterparts. Moreover, native distal femur fractures were also not further classified in the existing literature. To allow for a comparison of similar fracture configurations and corresponding treatment strategies, we focused on native Type A fractures, which are comparable to Lewis–Rorabeck Type I/II fractures as extra-articular injuries and are treated using the same surgical approach. Matching our data, two registry-based cohort studies by Mather et al. and Upfill-Brown et al. reported comparable mortality rates, with no significant differences between native and periprosthetic distal femur fractures [12, 13]. Conversely, data from the Danish National Patient Registry indicated poorer survival in patients over 60 years with native fractures compared to those with periprosthetic fractures [14]. This contrasts with the findings of Streubel et al., who reported higher mortality rates in patients with periprosthetic fractures versus those with native fractures in the same age group [15]. In patients over 60 years of age with distal femur fractures, 30-day mortality has been reported at approximately 2% for non-periprosthetic fractures and 8% for periprosthetic fractures [15]. Larsen et al. likewise reported 30-day mortality rates of 8% and 10% in geriatric patients [14]. In contrast, our study observed slightly lower inpatient mortality rates of 3.3% in native distal femur fractures and 6.1% across all periprosthetic distal femur fractures. However, these figures are only partially comparable, as a complete 30-day follow-up was not available in our cohort. Furthermore, our study included only surgically treated patients, whereas Larsen et al. demonstrated a significantly higher mortality risk in conservatively managed geriatric patients. An analysis from the National Trauma Data Bank of more than 26,000 patients reported an inpatient mortality of 8.3% for distal femur fractures, which is higher than in our series [16]. The comparatively lower mortality in our study may be explained by the more recent study period with corresponding improvements in perioperative management and the exclusion of non-surgically treated patients. These mortality rates highlight the high morbidity associated with this fracture type and emphasize the need for comprehensive perioperative care.

Postoperative complications were frequent, particularly in the native distal femur fracture group. This is in line with existing data in geriatric populations with systemic postoperative complication rates range from 38% to 49% [5, 6]. These findings underscore the potential benefit of adopting a multidisciplinary orthogeriatric co-management for distal femur fractures in the elderly to improve the postoperative outcomes [17]. Interestingly, higher morbidity was observed in native fractures, which may be attributed to distinct failure mechanisms. While native fractures generally occur in elderly, frail patients with advanced osteoporosis, periprosthetic fractures arise in the context of an existing implant, where mechanical factors such as stress concentration at the implant–bone interface are predominant [18, 19]. In our cohort, however, patients with native fractures were younger and had a lower comorbidity burden. In contrast, previous studies have reported higher rates of surgical site complications in periprosthetic fractures compared to native fractures, but no significant differences were found regarding revision surgery rates or blood transfusion requirements [13]. The mean operative time was approximately 140 min for both fracture types, consistent with prior reports for native distal femur fractures [12]. Although LOS tended to be shorter in patients with periprosthetic fractures, the overall LOS in our cohort was relatively long when compared to international data. For example, Hart et al. reported LOS values of 7.5 days after ORIF and 7.3 days after distal femoral replacement for the treatment of native distal femur fractures in a series of 38 patients [20], while Darrith et al. described similarly short stays for periprosthetic fractures [21]. These discrepancies likely reflect differences in healthcare systems, discharge planning protocols, and access to inpatient rehabilitation services. Supporting this, an Austrian study on geriatric distal femur fractures reported a mean LOS of 14.7 days, which is more comparable to our findings [5]. Another critical outcome is the loss of mobility. Several studies have demonstrated a substantial reduction in mobility following distal femur fractures [22, 23]. Our findings confirm this observation, with approximately half of patients with native distal femur fractures and more than one-third of those with periprosthetic fractures being immobile at discharge. These figures underscore the significant impact of these injuries on functional independence and quality of life, particularly in elderly and multimorbid populations. The results of this study suggest that patients with Lewis–Rorabeck Type I/II periprosthetic distal femur fractures, which are most commonly treated with ORIF, exhibit an early postoperative course during the inpatient stay that is comparable to that of patients with native distal femur fractures.

Lewis–Rorabeck Type III fractures, which involve a loose prosthesis and typically require revision arthroplasty, showed no differences in mobility at discharge and mortality compared to Lewis-Rorabeck Type I/II fractures in our study [24]. However, these patients experienced considerably longer delays to definitive surgery, and revision surgery was performed more frequently. Similar findings have been reported by Ruder et al. and Hoellwarth et al., who observed comparable functional outcomes and mortality rates in patients with periprosthetic distal femur fractures treated either with ORIF or DFR [23, 25]. In contrast to our findings, some studies suggest that DFR may be associated with shorter time to discharge, fewer surgery-related complications, and a reduced need for revision surgery compared with ORIF [26, 27]. A systematic review focusing on very distal periprosthetic femur fractures supports this assertion, indicating that DFR may allow for earlier full weight-bearing and lower revision surgery rates than ORIF [28]. The increased rate of revision surgeries in our study highlights the procedural complexity associated with revision arthroplasty. It is well established that surgical delays beyond 48 h are associated with increased mortality in patients with periprosthetic fractures [6, 29, 30]. Nevertheless, the timing of surgery should be interpreted with caution as a prognostic factor. In many elderly patients, early surgery is not always feasible due to medical instability or the need for optimization of multiple comorbidities before anesthesia and surgery can safely proceed [25]. Furthermore, the delay in the operation can be explained by the more complex surgical planning and provision of the corresponding components of the revision prostheses.

The distribution of fracture types in our cohort corresponds well with previously reported epidemiological patterns, with Lewis–Rorabeck Type II fractures being the most common configuration and Type III fractures occurring less frequently [24, 31]. Regarding surgical treatment, our results closely reflect current practice guidelines. The majority of native distal femur fractures and Lewis–Rorabeck Type I/II fractures were treated with ORIF, typically using locking plate systems. Intramedullary nailing was seldom used in our cohort. For Type III fractures, revision arthroplasty was the dominant approach, consistent with international consensus [9]. Hoellwarth et al. emphasized that treatment decisions should be guided by the fracture location and the quality of remaining bone stock [25]. Notably, even fractures involving the femoral component could achieve similar outcomes with both ORIF and DFR [25, 32]. Importantly, emerging evidence suggests that construct stability in distal femur fracture fixation can be significantly enhanced by dual plating techniques [33, 34]. While our study did not specifically analyze fixation techniques beyond the level of ORIF versus arthroplasty, this represents an important area for future investigation.

Several limitations should be acknowledged. As a retrospective, single-center study, the findings may be affected by selection and information bias, including potential misclassification of fractures and incomplete documentation of treatment decisions. Although multivariate comparisons were performed, residual confounding factors such as patient frailty and pre-injury functional status may still have influenced the results. Additionally, our analysis focused on the early postoperative period. While postoperative follow-ups are generally conducted at the center where surgery was performed, we experienced a substantial loss of follow-up data, with 47.4% of patients not returning for postoperative assessment. This is common in geriatric trauma populations with high comorbidity. Nevertheless, this considerable loss to follow-up may have led to an underestimation of revision surgery rates and limits the interpretation of postoperative outcomes beyond the inpatient period. Longer-term follow-up would be valuable to provide a more comprehensive understanding of clinical outcomes and functional recovery. Furthermore, as a Level I trauma center, several different surgeons performed the procedures, which may affect the generalizability of the results. Finally, although changes in clinical practice and implant design over time could influence treatment approaches and outcomes, the relatively short study period limits the potential impact of these factors.

## Conclusion

This study compares periprosthetic distal femur fractures with native distal femur fractures based on implant stability. Our findings challenge the common perception that periprosthetic distal femur fractures are inherently more difficult to manage or are linked to higher rates of early complications. When the prosthesis is stable, Lewis–Rorabeck Type I/II periprosthetic fractures can be treated similarly to native fractures, achieving identical clinical results. Lewis–Rorabeck Type III fractures, which require revision arthroplasty due to implant loosening, were associated with longer waiting times to surgery and a higher frequency of revision procedures, illustrating the complexity and clinical burden of these cases. Nevertheless, they demonstrated a comparable early clinical course in terms of early mobility, complications, and inpatient mortality.

## Supplementary Information

Below is the link to the electronic supplementary material.

Supplementary file1 (DOCX 23 KB)

## Data Availability

The datasets generated and analysed during the current study are not publicly available due to institutional and ethical restrictions related to patient confidentiality and data protection laws. Anonymized data may be available from the corresponding author on reasonable request and with approval from the local ethics committee.

## References

[1] Gausden EB et al (2024) What’s new in periprosthetic femur fractures? J Arthroplasty 39(9s2):S18–s25 38642853 10.1016/j.arth.2024.04.037

[2] Epidemiology (2022) *and* Characteristics of femoral periprosthetic fractures: data from the characteristics, outcomes and management of periprosthetic fracture service evaluation (COMPOSE) cohort study. Bone Joint J, 104–b(8): p. 987–996 10.1302/0301-620X.104B8.BJJ-2021-1681.R1 35909377

[3] Lindahl H (2007) Epidemiology of periprosthetic femur fracture around a total hip arthroplasty. Injury 38(6):651–654 17477925 10.1016/j.injury.2007.02.048

[4] Konan S et al (2016) Periprosthetic fractures associated with total knee arthroplasty: an update. Bone Joint J, 98–b(11): p. 1489–1496 10.1302/0301-620X.98B11.BJJ-2016-0029.R1 27803224

[5] Kammerlander C et al (2012) Functional outcome and mortality in geriatric distal femoral fractures. Injury 43(7):1096–1101 22405338 10.1016/j.injury.2012.02.014

[6] Moloney GB et al (2016) Geriatric distal femur fracture: are we underestimating the rate of local and systemic complications? Injury 47(8):1732–1736 27311551 10.1016/j.injury.2016.05.024

[7] Bottle A et al (2020) Periprosthetic fractures: the next fragility fracture epidemic? A National observational study. BMJ Open 10(12):e042371 33303466 10.1136/bmjopen-2020-042371 PMC7733197

[8] Lampert C et al (2024) *Open Reduction and Internal Fixation Is a Feasible Alternative to Femoral Revision Arthroplasty in Geriatric Patients with Vancouver B2/3 Type Periprosthetic Fractures: A Study Analyzing In-Hospital Outcomes.* J Clin Med, 13(21) 10.3390/jcm13216475 PMC11546701 39518614

[9] Rorabeck CH, Taylor JW (1999) Periprosthetic fractures of the femur complicating total knee arthroplasty. Orthop Clin North Am 30(2):265–277 10196428 10.1016/s0030-5898(05)70081-x

[10] Healy WL, Siliski JM, Incavo SJ (1993) Operative treatment of distal femoral fractures proximal to total knee replacements. J Bone Joint Surg Am 75(1):27–34 8419387 10.2106/00004623-199301000-00005

[11] Johnston AT et al (2012) Periprosthetic fractures in the distal femur following total knee replacement: A review and guide to management. Knee 19(3):156–162 21741844 10.1016/j.knee.2011.06.003

[12] Mather AM et al (2023) Primary and periprosthetic distal femur fractures in older adults: no difference in 12-Month mortality and Patient-Reported outcomes. J Orthop Trauma 37(10):492–499 37296087 10.1097/BOT.0000000000002649

[13] Upfill-Brown A et al (2023) Short-term outcomes of periprosthetic compared to native distal femur fractures, a National database study. Arch Orthop Trauma Surg 143(1):115–124 34185154 10.1007/s00402-021-04000-0

[14] Larsen P, Ceccotti AA, Elsoe R (2020) High mortality following distal femur fractures: a cohort study including three hundred and two distal femur fractures. Int Orthop 44(1):173–177 31081515 10.1007/s00264-019-04343-9

[15] Streubel PN et al (2011) Mortality after distal femur fractures in elderly patients. Clin Orthop Relat Res 469(4):1188–1196 20830542 10.1007/s11999-010-1530-2 PMC3048257

[16] Tsai SHL et al (2021) Distal femur fractures have a higher mortality rate compared to hip fractures among the elderly: insights from the National trauma data bank. Injury 52(7):1903–1907 33896612 10.1016/j.injury.2021.04.023

[17] Prestmo A et al (2015) Comprehensive geriatric care for patients with hip fractures: a prospective, randomised, controlled trial. Lancet 385(9978):1623–1633 25662415 10.1016/S0140-6736(14)62409-0

[18] Chuluunbaatar Y et al (2024) Early and 1-year mortality of native geriatric distal femur fractures: A systematic review and time-to-event meta-analysis. J Clin Orthop Trauma 50:102375 38495682 10.1016/j.jcot.2024.102375 PMC10943051

[19] Fleischman AN, Chen AF (2015) Periprosthetic fractures around the femoral stem: overcoming challenges and avoiding pitfalls. Ann Transl Med 3(16):234 26539451 10.3978/j.issn.2305-5839.2015.09.32 PMC4598449

[20] Hart GP et al (2017) Open reduction vs distal femoral replacement arthroplasty for comminuted distal femur fractures in the patients 70 years and older. J Arthroplasty 32(1):202–206 27449717 10.1016/j.arth.2016.06.006

[21] Darrith B et al (2020) Periprosthetic fractures of the distal femur: is open reduction and internal fixation or distal femoral replacement superior? J Arthroplasty 35(5):1402–1406 31924488 10.1016/j.arth.2019.12.033

[22] Hoffmann MF et al (2012) Outcome of periprosthetic distal femoral fractures following knee arthroplasty. Injury 43(7):1084–1089 22348954 10.1016/j.injury.2012.01.025

[23] Ruder JA et al (2017) Predictors of functional recovery following periprosthetic distal femur fractures. J Arthroplasty 32(5):1571–1575 28131543 10.1016/j.arth.2016.12.013

[24] Schreiner AJ et al (2018) Adverse events in the treatment of periprosthetic fractures around the Knee - a clinical and radiological outcome analysis. Z Orthop Unfall 156(3):287–297 29342496 10.1055/s-0043-123831

[25] Hoellwarth JS et al (2018) Equivalent mortality and complication rates following periprosthetic distal femur fractures managed with either lateral locked plating or a distal femoral replacement. Injury 49(2):392–397 29208310 10.1016/j.injury.2017.11.040

[26] Ebraheim NA et al (2015) Periprosthetic distal femur fracture after total knee arthroplasty: A systematic review. Orthop Surg 7(4):297–305 26790831 10.1111/os.12199 PMC6583744

[27] Saidi K et al (2014) Supracondylar periprosthetic fractures of the knee in the elderly patients: a comparison of treatment using allograft-implant composites, standard revision components, distal femoral replacement prosthesis. J Arthroplasty 29(1):110–114 23680503 10.1016/j.arth.2013.04.012

[28] Rubinger L et al (2021) Very distal femoral periprosthetic fractures: replacement versus fixation: A systematic review. J Orthop Trauma 35(11):573–583 33993176 10.1097/BOT.0000000000002080

[29] Bhattacharyya T et al (2007) Mortality after periprosthetic fracture of the femur. J Bone Joint Surg Am 89(12):2658–2662 18056498 10.2106/JBJS.F.01538

[30] Zuckerman JD et al (1995) Postoperative complications and mortality associated with operative delay in older patients who have a fracture of the hip. J Bone Joint Surg Am 77(10):1551–1556 7593064 10.2106/00004623-199510000-00010

[31] Makaram NS et al (2022) Reliability of current classification systems for periprosthetic distal femur fractures. Injury 53(10):3430–3437 35948511 10.1016/j.injury.2022.08.002

[32] Kim W, Song JH, Kim JJ (2015) Periprosthetic fractures of the distal femur following total knee arthroplasty: even very distal fractures can be successfully treated using internal fixation. Int Orthop 39(10):1951–1957 26300375 10.1007/s00264-015-2970-9

[33] Kriechling P et al (2024) Double plating is a suitable option for periprosthetic distal femur fracture compared to single plate fixation and distal femoral arthroplasty. Bone Jt Open 5(6):489–498 38862133 10.1302/2633-1462.56.BJO-2023-0145.R1 PMC11166487

[34] Sanders R et al (1991) Double-plating of comminuted, unstable fractures of the distal part of the femur. J Bone Joint Surg Am 73(3):341–346 2002071

