# Supplementary material for: Challenging the myth: comparing early complications of native and periprosthetic distal femur fractures. The role of implant stability
Source: Arch Orthop Trauma Surg. 2026 Jan 7;146(1):30. doi: 10.1007/s00402-025-06173-4 (PMC12779657; doi:10.1007/s00402-025-06173-4)
Supplement: Supplementary file 1 — Supplementary file1 (DOCX 23 KB) [file 402_2025_6173_MOESM1_ESM.docx]

Supp. 1: Analysis of Lewis-Rorabeck Type I/II and native distal femur fractures Type A-C on perioperative parameters and early complications

|  | Native distal femur fracture (n = 90) | Lewis-Rorabeck Type I/II (n = 49) | β/OR | p-value |  |  |
| --- | --- | --- | --- | --- | --- | --- |
| Mean ± SD | | | |  |  |  |
| Duration of surgery (minutes) | 135.66 ± 54.37 | 148.02 ± 72.72 | -11.49 | 0.351 |  |  |
| Time to surgery (hours) | 47.48 ± 70.46 | 30.74 ± 29.98 | 1.35 | **0.896** |  |  |
| Hospital stay (days) | 15.41 ± 11.67 | 13.29 ± 5.58 | 1.42 | 0.455 |  |  |
| Percent of cases (n) | | | |  |  |  |
| Patients with ≥ 1 non-surgical complication | 21.11% (19) | 14.29% (7) | 2.38 | 0.114 |  |  |
| Inpatient mortality | 3.33% (3) | 4.08% (2) | 2.91 | 0.386 |  |  |
| Patients receiving blood transfusion | 17.78% (16) | 28.57% (14) | 0.64 | 0.371 |  |  |
| Revision surgery performed | 6.67% (6) | 6.12% (3) | 1.22 | 0.808 |  |  |
| Walking ability at discharge | 60.00% (54) | 61.22% (30) | 1.83 | 0.167 |  |  |

n numbers of observation; β Regression Coefficient; OR Odds Ratio; SD standard deviation; All models were adjusted for sex, age, BMI and ASA Score. Values are presented as mean ± standard deviation or percentage unless otherwise indicated. Significant p values are in bold.
